# A remote healthcare monitoring framework for diabetes prediction using machine learning

**DOI:** 10.1049/htl2.12010

**Published:** 2021-05-02

**Authors:** Jayroop Ramesh, Raafat Aburukba, Assim Sagahyroon

**Affiliations:** ^1^ Computer Science and Engineering American University of Sharjah Sharjah United Arab Emirates

## Abstract

Diabetes is a metabolic disease that affects millions of people each year. It is associated with an increased likelihood of vital organ failures and decreased quality of life. Early detection and regular monitoring are crucial for managing diabetes. Remote patient monitoring can facilitate effective intervention and treatment paradigms using current technology. This work proposes an end‐to‐end remote monitoring framework for automated diabetes risk prediction and management, using personal health devices, smart wearables and smartphones. A support vector machine was developed for diabetes risk prediction using the Pima Indian Diabetes Database, after feature scaling, imputation, selection and augmentation. This work achieved the performance metrics of accuracy, sensitivity and specificity scores at 83.20%, 87.20% and 79% respectively through the tenfold stratified cross validation method, which is competitive with existing methods. Patients can use multiple healthcare devices, smartphones and smartwatches to measure vital parameters, curb the progression of diabetes and close the communication loop with medical professionals. The proposed framework enables medical professionals to make informed decisions based on the latest diabetes risk predictions and lifestyle insights while attaining unobtrusiveness, reduced cost, and vendor interoperability.

## 1 INTRODUCTION

Diabetes is a metabolic disorder that causes a lack or resistance to insulin, which is a hormone critical for the regulation of blood sugar levels. The common symptoms are polyuria, polydipsia, rapid weight loss, vision blurriness and fatigue [1]. This leads to severe complications like strokes, blindness, miscarriages and organ failures. The World Health Organization in 2014 reported that approximately 422 million people worldwide have diabetes, and estimates 1.6 million deaths directly caused by the disease [2]. Medical guidelines recommend the need for early diagnosis to identify risk‐prone people and for patients to proactively self‐monitor their lifestyle to mitigate risk factors [3]. Remote patient monitoring (RPM) can help prevent the staggering number of deaths caused by diabetes by means of early detection and timely warnings to the patient and medical professionals. RPM reduces the need for regular checkups, gauge continuous treatment effectiveness and allow intervention techniques for alerting professionals, thereby enhancing the quality of life for patients.

Traditional methods of chronic disease management use rule‐engines or score estimations for diabetes risk identification, which are not as effective as machine learning techniques in detecting patient health conditions [4]. Recent developments in machine learning and feature engineering [5], [6], present new possibilities to improve early diagnosis and treatment outcomes of chronic diabetes patients. Recent studies in diabetes management presented the most common types of systems, which are insulin self‐management apps, wearable blood glucose monitors, automated SMS risk alerts and virtual diagnostician coaching [3]. Su [7] surveyed methods in literature to prove the efficacy of RPM techniques for diabetes management, and showed that self monitoring and regular intervention led to reduction of patient glucose levels. One of the major limitations in existing systems arises from isolated diabetes prediction models that do not interact with real patients or their data. Notably, there is also a lack of unified healthcare device vendor‐independent frameworks that can obtain multi‐faceted patient readings to enable easy monitoring and management [8].

The American Diabetes Association (ADA) states the frequency of personal health device use is one of the most important factors for lowering diabetes risks in patients [9]. Vhaduri et al. [10], reinforce the argument that continuous glucose monitoring with personal health devices in diabetes management can aid in early diagnosis. In their study, they discuss the importance data driven adherence analysis in algorithms to positively affect behaviours of chronic diabetic patients. Regular patient adherence to personal health devices or smart wearables is proportional to their perceived value of observable insights from the collected data. Merely viewing the accumulated information on a portal is not sufficient for the patients to continue using these devices. By incorporating medical professionals into the system, where they also receive timely and accurate alerts about the patients' state, actionable decisions can be made to improve disease outcomes. This reveals the importance of the monitoring devices, and the pertinence of data driven predictions in healthcare contexts. There is an increasing volume of physiological data that is being collected from off‐the‐shelf wearables and general purpose smartphones by entities such as Google, Apple and Fitbit. The growing popularity of mainstream wearable technology like smartwatches, fitness trackers and wrist bands can enable the transition of RPM from being used solely in ambulatory settings, to regular environments.

This work proposes an automated smart health monitoring framework for diabetes prediction and management by the implementation of supervised machine learning (ML) algorithms and seamless integration of multiple healthcare devices and consumer devices. The system makes live predictions based on the patient vital signs such as blood glucose and blood pressure to notify medical professionals for intervention. The medical professionals are provided holistic view of the patient lifestyle metrics such as physical activity levels and caloric expenditure through a dashboard to assist them in making informed decisions for diabetes treatment and management. Only minimal patient‐side interaction is needed in terms of setting up, as they only have to use their healthcare devices and the proposed framework can automatically retrieve vital data and provide predictions.

The main contributions of this paper are mentioned as follows:

- Develop an automated remote monitoring framework that consolidates patient vital data from different multi‐vendor personal health devices, smartphones and smartwatches to enhance diagnostic decision making.
- Integrate a machine learning model within framework and use a subset of the data collected to provide live diabetes risk predictions.
- Leverage the ability of machine learning predictions to generate timely alerts for medical professionals and provide associated vital patient data.

This paper is organised as follows, Section 2 presents existing approaches, Section 3 discusses the methodology of ML algorithms, Section 4 describes the implementation of the proposed framework, Section 5 presents the results and discussion of ML models, and Section 6 concludes this work.

## 2 LITERATURE REVIEW

Malasinghe et al. [1] surveyed the most recent glucose monitoring methods and reported that closed‐loop systems where the feedback mechanism provided immediate intervention to patients were the most effective in diabetes control and management. It was also noted that most existing frameworks were unable to combine multiple devices together, which introduces concerns of privacy and interoperability when new devices need to be added or health readings need to be obtained. Aburukba et al. [11] proposed a brokering framework for RPM that integrates multiple cloud health providers and utilised a custom rule engine to provide timely intervention to patients. This study addressed the limitations caused by healthcare device vendor interoperability concerns and data security to integrate the user vital measurements such as blood glucose, blood pressure and blood oxygen saturation from multiple devices operated by different vendors into a unified platform.

Chatrati et al. [12] presented a smart home health monitoring system that uses patient blood pressure and glucose readings at home and notifies healthcare providers if abnormalities in hypertension and diabetes levels are detected. They leveraged a combination of conditional decision‐making, and ML approaches such as support vector machines (SVM), k‐nearest nNeighbours (k‐NN), decision tree (DT), logistic regression (LR) and discriminant analysis (DA) to analyse the manual patient measurement inputs into the system. The algorithms achieved accuracy measures of 75%, 74%, 66.1%, 74.5% and 74.7% respectively. Sonar et al. [13] developed four algorithms: DT, naive Bayes (NB), SVM and artificial neural networks for the prediction of diabetes, and achieved accuracy measures of 74%, 80%, 82% and 82% respectively. Al Zebari [14] et al. utilised a series of ML methods for prediction of diabetes such as DT, LR, DA, SVM, k‐NN and their variants. tenfold cross‐validation was used for evaluation and the average prediction accuracy measures were recorded. The best performing method was LR with an accuracy score of 77.7% and the poorest performing method was coarse Gaussian SVM. Saha [15] implemented ML techniques such as neural network (NN), SVM, and Random Forest for diabetes prediction. Preprocessing methods of imputation, scaling, data normalisation and principle component analysis was applied. The NN was the best performing model with configuration parameters of 100 epochs and ten batch size. It achieved an accuracy measure of 80.4%. Wei [16] employed a Deep NN for the prediction of diabetes and attained an overall accuracy measure of 77.86%. Scaling was applied for preprocessing, and the configuration parameters of ReLU activation function, Nadam optimiser, 100 epochs and ten batch size were used. tenfold cross validation technique was also applied for evaluation.

There are a few promising smart healthcare monitoring frameworks focusing on relating machine learning techniques with physiological data, to actively improve health outcomes through recommendations or alerting medical professionals. Ali et al. [17] presents an ensemble deep learning model that automatically recommends a diet plan or activities according to the condition of a patient suffering from chronic heart disease. They used a feature fusion method to combine patient physiological data from wearable sensors and electronic medical records. Recently, it has been shown that even patient social media posts in conjunction with sentiment analysis can be used for the early detection of diseases, primarily mental health [18]. In [19], smartphones, wearable sensors, social networks and electronic medical records have been used to develop a prewarning system for various diseases such as diabetes, mental health and blood pressure,. This system relies on Big Data analytic engines based on data mining, ontologies, and deep learning to classify the health conditions. Ontologies can be used provide semantic knowledge about concepts in a particular domain to data driven solutions. In [20], a type 2 fuzzy ontology aided recommendation system was implemented to recommend medical prescriptions and diet recommendations by estimating patient risk factors using wearable sensors.

The proposed framework combines multiple ideas partially present in other studies, and incorporates the element of user‐friendliness by requiring minimal additional input from the patient. Most existing works focus on collecting vital patient information using multiple sensors and limited medical devices or predicting diabetes outcomes from electronic medical records and public datasets. Machine learning models developed to this end have considerably high performance, but they are limited as the models are never tested in real‐life scenarios with real patient data. It is also observed that stand‐alone sensors, or custom devices are used to acquire physiological data from patients. In real scenarios, it is not practical to equip patients with multiple sensors and custom wearables for continuous monitoring. Patients would typically utilise multiple healthcare devices, often manufactured by different vendors to measure vital signs. To the best of the authors' knowledge, there is a clear need for a system that can scale horizontally without interoperability concerns between different vendor devices, while providing the intelligence required for early diabetes risk detection. With the proposed framework in this work, the wealth of data obtained from personal health devices and consumer wearables can be used to generate machine learning based diabetes risk predictions.

## 3 METHODOLOGY

**FIGURE 1:**
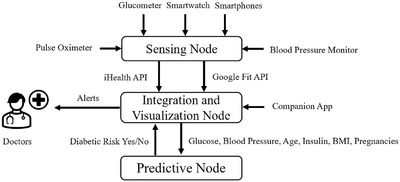
The components of the smart healthcare framework

The proposed framework aims to consolidate patient data from personal health devices and consumer wearables, provide early detection of diabetes risk, and keep medical professionals updated with the latest information. The components of the system and their flow are presented in Figure 1.

The static user profile information obtained are height, weight, age, and gender. The continuously monitored user vitals obtained are heart rate, blood oxygen, perfusion index, systolic blood pressure (mmHG), diastolic blood pressure (mmHg), glucose level (mg/dL), medication status (pills taken/pills not taken), step count, calories, total activity duration and types of activity performed. A mobile application was developed to act as a gateway to consolidate the one‐time authentication information for the different vendor cloud services, and transmit this information to the server. The primary advantage of the proposed system is that user do not have to enter their user data explicitly as the mobile application directly connects to the respective vendor cloud platforms and extracts the vitals measured by the devices. The vendor cloud services in this work are Google Fit [37], which stores the data acquired from the smartphone, smartwatch and iHealth API [36], which stores the data acquired from the iHealth medical devices. An API is an Application Programming Interface that enables communication between different software intermediaries and end‐devices. The server visualises the user vitals obtained from the multiple devices on a medical professionals or caregiver's web portal, and also provides preliminary intelligent predictions about the diabetic condition of the user.

The nodes that constitute the system are listed below, and described further in Section 4.

- Sensing node: Physiological patient data is acquired from personal health devices, smartphones and smartwatches.
- Integration and visualisation node: Patient data from multiple sources are consolidated and made accessible through a web portal.
- Prediction node: A machine learning model generates diabetes predictions using a subset of the patient data collected.

**FIGURE 2:**
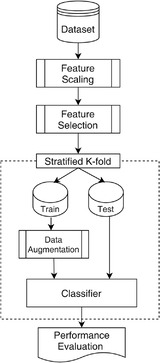
Framework of the machine learning pipeline

The proposed pipeline for the implementation of machine learning models in the prediction node is depicted in Figure 2. First, imputation is performed to account for missing data, and this is followed by feature scaling to standardise the range of the dataset values. Feature selection methods are applied to remove redundant features that do not contribute highly to the prediction outcome during training and enhance overall model fidelity. To rectify class imbalances, an oversampling method is used to synthesize strongly similar samples of the minority class in the data augmentation step. Finally, the binary classifiers are fit with the data during the *k*‐fold cross validation step, where all samples are used for both training, validation and testing, thereby producing a more robust classifier.

### 3.1 Dataset

The dataset used in this work is the PIMA Indian Diabetes Database accessed from the University of California, Irvine ML repository [21]. The dataset has a sample size of n=768 patients, each patient with nine feature attributes. The patients are all females between the ages of 21 to 81 and their data was collected by the National Institute of Diabetes, Digestive and Kidney Diseases. The eight continuous numeric attributes are number of pregnancies, glucose level (mg/dL), diastolic blood pressure (mmHg), skin fold thickness (mm), body mass index (BMI), serum insulin level (mU/mL), age (years) and a diabetes hereditary factor pedigree function. The final attribute is the target variable that denotes the diabetes prediction class, where a categorical value of 1 indicates presence of diabetes, and the categorical value of 0 indicates the absence of diabetes.

### 3.2 Feature scaling

Out of the 768 patient samples, five had a glucose level of 0, 11 had a BMI of 0, 28 had a diastolic blood pressure of 0, 192 had a skin thickness of 0 and 140 had an insulin level of 0. Domain knowledge concurs that it is not possible for patients to have 0 values in these physiologically pertinent variables. To ensure the randomly missing values do not affect the quality of the patterns mined by the ML models, a suitable imputation technique must be used [22].

With randomised clinical trial datasets, the multivariate imputation by chained equations (MICE) method [23] has been shown to be highly efficient for handling missing data, under the general assumption of the data being either missing at random, or missing completely at random [24]. MICE iteratively estimates values for missing data for each specified feature using the other corresponding features as predictors. A strong correlation between the features of a dataset, and a robust prediction strategy allows for better imputed value estimates. In this work, the MICE approach was utilised with a decision tree regression model to impute missing values and convergence was reached after five iterations.

Standardisation is a scaling technique where the values are centred around the mean with a unit standard deviation. This means that the mean is 0 and the distribution has a unit standard deviation. Since outliers will not be affected by standardisation, no outliers are lost. This aids in faster convergence during training [22].

### 3.3 Feature selection

Feature selection reduces the computational complexity of prediction algorithms by the removal of non‐informative or redundant input features from the dataset. This reduces the uncertainty in prediction and increases the overall effectiveness of the model.

In this work, three types of feature selection techniques are considered and tested for each prediction model, which are the chi‐squared test, extremely randomised trees classifier (extra trees), and least absolute shrinkage and selection operator (LASSO).

Chi‐squared test is a non parametric statistical measure to test the correlation between two variables [25]. The method generates a value that quantifies the dependence between the input features and the output to be predicted. The higher the value, the stronger is the correlation between the input feature and output feature, and features with values lower than a critical value is discarded. The numerical values of the features in this dataset were discretised based on their frequency of occurrence, as the chi squared method performs on categorical data.

Extra trees fits a number of randomised decision trees on the various subsets of the entire dataset [26]. The input variables and cut‐off values are selected randomly to split a node in the tree construction process, such that they are completely independent from the output variable. Each tree leads to a different model, trained with subsets of features, and the algorithm ranks the importance of the contributing features according to a threshold measure called the Gini index.

LASSO is a L1 regularisation technique used for feature selection to simplify the interpretation of the dataset [27]. In this method, regression analysis is performed to estimate parameters and select models simultaneously, thereby reducing the variability of the features by shrinking coefficients of the non‐correlating features to 0.

Table 1 presents the most important features selected by each of the methods respectively, with their ranking measures. The chi‐squared test uses the chi‐score, Extra trees uses the Gini index, and LASSO uses coefficients of regression. The features of glucose, insulin, BMI and age consistently ranked high in importance and were selected by each of the applied techniques. After experimentation with the features, it was found the removal of skin fold thickness and diabetes pedigree features improved the overall performance of the model.

**TABLE 1:** Feature selection values obtained for the dataset

**Features**	**Chi‐squared test**	**Extra trees**	**LASSO**
# of pregnancies	111.52	0.102	0.0
**Glucose**	**1437.40**	**0.220**	**0.0062**
Blood pressure	53.66	0.087	0.0
Skin fold thickness	143.69	0.092	0.0
**Insulin**	**6882.18**	**0.137**	**0.0003**
**BMI**	**109.12**	**0.128**	**0.0005**
**Age**	**181.30**	**0.125**	**0.0002**
Diabetes pedigree	5.39	0.106	0.0

### 3.4 Data augmentation

The dataset was shown to have data imbalances, with the majority of samples belonging to the non‐diabetes class at n=268. The number of diabetes class samples were n=500. To reduce the biases in the created models, the synthetic minority over‐sampling technique (SMOTE) is used [28]. SMOTE is an oversampling technique that increases the number of minority class samples in the dataset, by generating new samples from existing minority class samples. The approach generates new minority class samples that are not duplicates, but convex combinations of two or more randomly chosen neighbouring data samples in the feature space. The application of SMOTE to clinical datasets improve model performance by reducing the negative effects of imbalanced data is observed in recent literature [29]. SMOTE is only applied on the training/validation split of the data samples so that the model sees equal numbers of both class types, and the test split is not modified. The number of samples of the non‐diabetes class after augmentation is n=500.

### 3.5 Machine learning

The ML algorithms implemented for binary prediction of the two outcomes diabetes and non‐diabetes, are the k‐NN, LR, Gaussian NB, and SVM‐RBF.

#### 3.5.1 k‐nearest neighbours

k‐NN [30] is defined a lazy, non‐parametric supervised learning algorithm that stores all dataset instances and classifies new unknown instances by majority vote of their k nearest instance neighbours. This algorithm has a high convergence speed and simplicity. A similarity measure, such as distance in an n‐dimensional space between each instance decides which class the instance is assigned to. The most popular similarity measure used is the Euclidean distance between two instances. If k=2, then an instance is assigned to the class of two of its nearest neighbours, where the computed Euclidean distances between the instances are the lowest. Equation (1) denotes the Euclidean distance between two points $x_{i}$ and $x_{j}$ and N is the total number of data instances. In this work, k−20 was selected through experimentation to yield the best performance.

(1) $$d\left(x_{i},x_{j}\right)=\sqrt{\sum_{i=1}^{N}{\left(x_{i}-x_{j}\right)}^{2}}$$

#### 3.5.2 Logistic regression

LR [31] is a statistical analysis technique in which one or more independent input variables predicts the probability of occurrence of a binary output variable. It is an extended case of the classic linear regression where there is one independent variable and one dependent variable. The algorithm estimates the relationship between the input and output variables by means of a best fit model and is described by Equation (2).

(2) $$P\left(y|x,w\right)=\frac{1}{1+\exp(-y\left(w^{T}x+b\right))}$$

Given data x, and weights w,b, the probability model defines y as the prediction class outcome, where $y_{i}\in 0,1$ indicates absence or presence of diabetes. If total number of training instances are N, then $x_{i}$ is the *i*th input instance and the optimum weights of w,b are found by minimising the negative log‐likelihood function described by Equation (3).

(3) $$\min_{w,b}\sum_{i=1}^{N}\log(1+\exp\left(-y_{i}\left(w^{T}x_{i}+b\right)\right)\;$$

#### 3.5.3 Gaussian Naive‐Bayes

NB [32] is a prediction technique based on the Bayes theorem of probability and assumes strong, or naive, independence between the feature attributes of the data. The Bayes theorem defined by Equation (4) is used to find the probability of an output prediction category using the input feature attributes for $x_{N}$ instances in the training set. P(A|B) is the probability of event **A** given event **B** has occurred, P(B|A) is probability of event **B** given event **A** has occurred, and P(A) and P(B) is probability for events **A** and **B** respectively.

(4) $$P\left(A|B\right)=\frac{P(B|A)\cdot P(A)}{P(B)}\;$$

In this work, the feature values are continuous data, hence a Gaussian distribution is applied. This is shown by Equation (5), where p is the probability, μ and σ are the parameterising mean and variance of all the quantitative attributes, $X_{i}$ and $x_{i}$ are the *i*th attribute and value of all the N data instances, C is the class label and $C_{j}$ is the outcome of presence of diabetes or absence of diabetes.

(5) $$p\left(X_{i}=x_{i}|C=c_{j}\right)=\frac{1}{\sqrt{2\pi \sigma_{i}^{2}}}\exp\left(-\frac{{\left(X_{i}-\mu_{ij}\right)}^{2}}{2\sigma_{ij}^{2}}\right)$$

#### 3.5.4 Support vector machines

SVM [33] is a supervised ML algorithm that performs prediction by optimally separating the data instances linearly with the help of support vectors. In binary prediction, the algorithm plots each data instance as a point in n‐dimensional space, where n is the number of feature attributes, and creates two maximum‐margin hyperplanes which provides the largest separation between the training instances of the identified classes. The data instances closest to the hyperplanes are called support vectors, and new instances are assigned to a class based on their relative distance to the hyperplanes. To predict outcomes of non‐linear data with SVM by mapping input features into a higher‐dimensional feature space, the radial basis kernel [33] is applied in this work to yield the SVM‐RBF. Equation (6) defines the minimising function that is used to find the optimum hyperplane margins and its constraints. w is a normal, b is a threshold and $x_{i}$ is the feature vector corresponding to each data instance.

(6) $$\begin{array}{c} \min_{w,b}\frac{1}{2}{∥\vec{w}∥}^{2}\\ \text{subject}\;\text{to}\;\text{the}\;\text{constraints}\\ y_{i}\left(\vec{w}\cdot {\vec{x}}_{i}+b\right)\geq 1 \end{array}$$

## 4 IMPLEMENTATION

This section discusses the prototype implementation of the RPM framework that deploys live ML models for diabetes prediction and providing intervention to users and medical professionals. Since the focus of this work is on integrating a diabetes prediction model and leveraging user acquired data to make predictions for enabling lifestyle management, the proposed system is based on the cloud based brokering framework that integrates multiple health cloud platforms and devices, a system introduced by Aburukba et al. [11]. The devices used in this work are the Samsung Note 8 smartphone [34], a Huawei GT 2 smartwatch [35], iHealth blood pressure monitor, iHealth glucometer and iHealth pulse oximeter [36].

**FIGURE 3:**
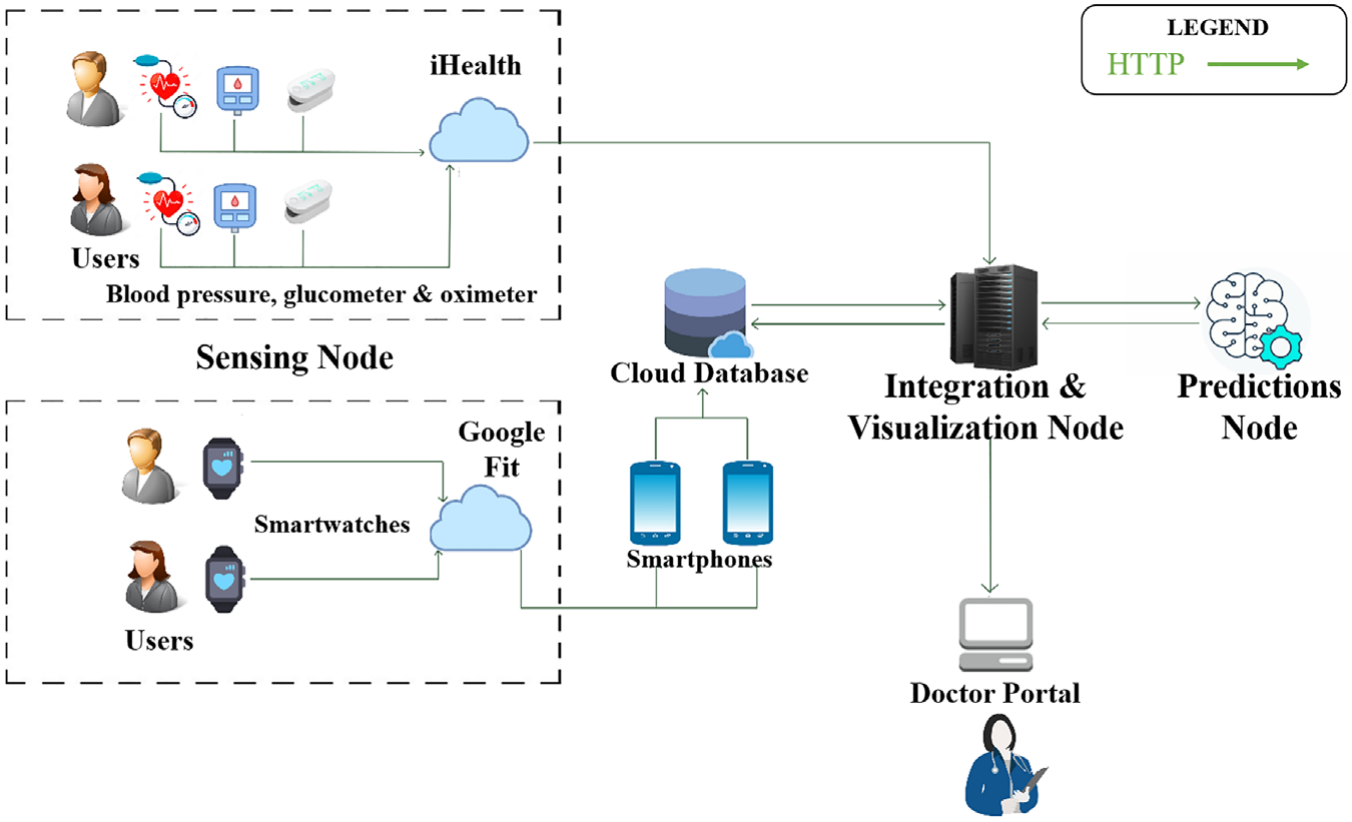
Architecture of proposed framework with devices, components and entities

Figure 3 shows the high‐level architecture of the platform, where user data is integrated from smartwatches and medical devices via a smartphone and transmitted to a RESTful server.

### 4.1 Sensing node

**FIGURE 4:**
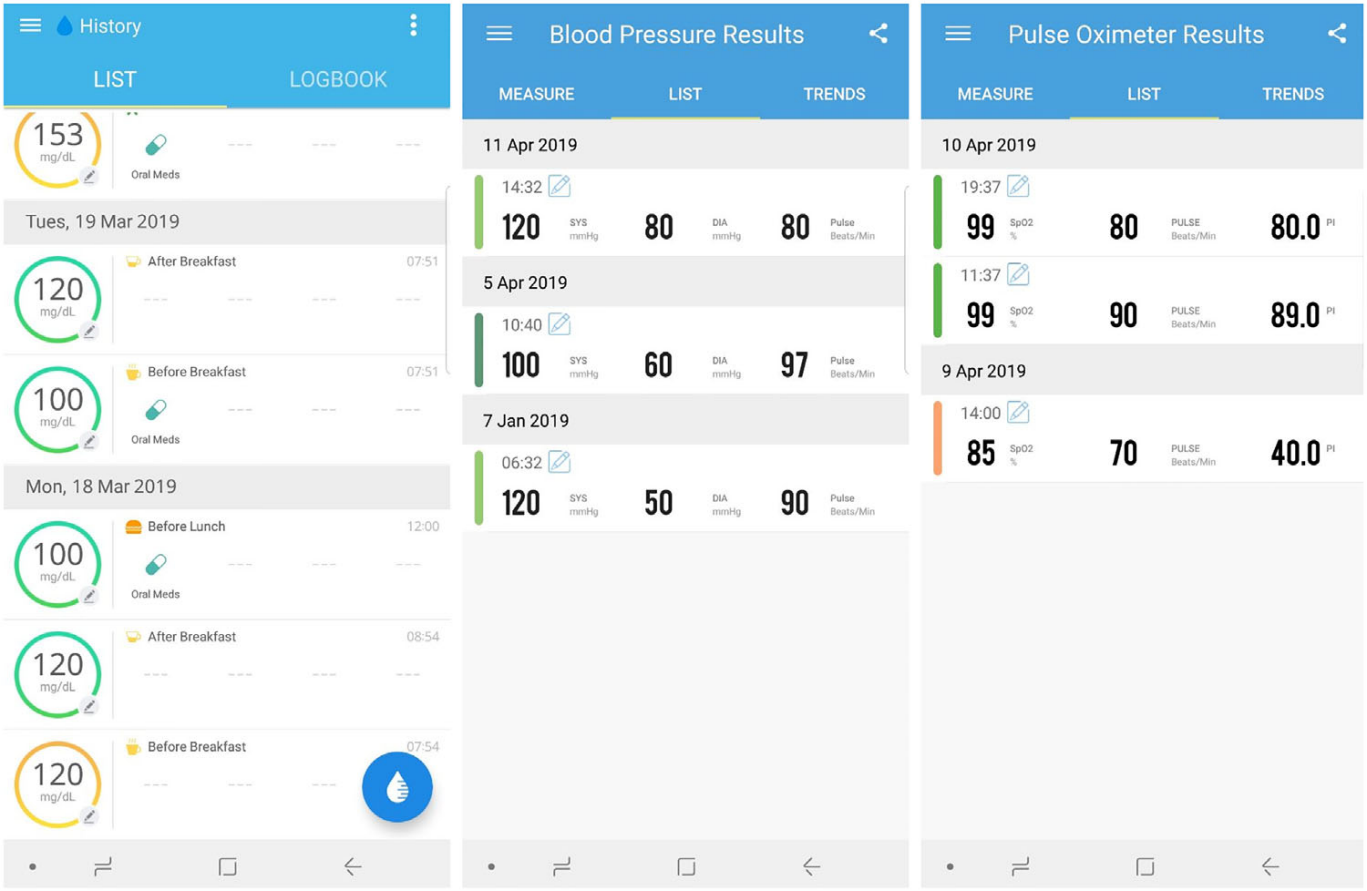
Patient data collected by the three iHealth devices as shown on their respective applications

This node comprises of the devices that actively and passively collects user data. The user initially inputs their static profile information. Active collection is enabled by the iHealth devices, where the user measures their vitals of glucose levels, blood pressure and blood oxygen saturation. Passive collection is enabled by the smartwatch and the smartphone, where the smartphone automatically tracks their steps, caloric expenditure, and activity type and duration. The iHealth vitals are automatically synced with the iHealth vendor cloud when a user takes a measurement using the glucometer, blood pressure monitor or pulse oximeter, and users can access this information by signing into their respective vendor‐specific web portal or mobile application. The data collected from each of the iHealth devices, for a single volunteer subject in this work is presented in Figure 4.

**FIGURE 5:**
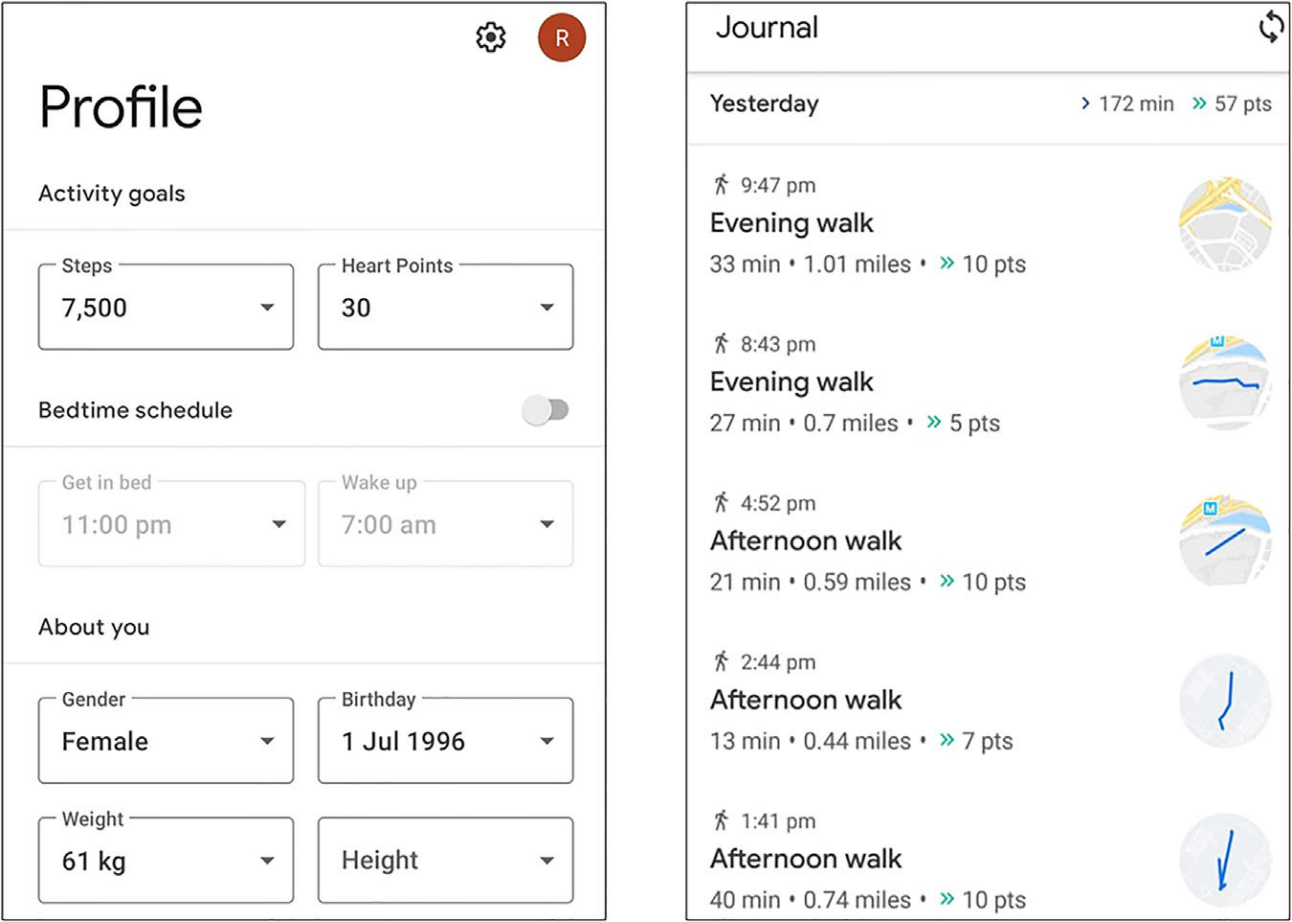
Patient data collected from smartphone/smartwatch by Google Fit as shown on the respective application

Smartphones and smartwatches with Android operating system utilises the available device sensors on each particular device to automatically collect and aggregate activity data and shows the fitness history to users via the Google Fit web portal or mobile application. The Google vendor API leverages the sensors of gyroscope and accelerometer for both smartphones and smartwatches, and the additional heart rate sensor on smartwatches to track movements, activities, heart rates and also estimates calories burned and count of steps. The data tracked by the Google Fit application for the subject in this work is presented in Figure 5.

The server in the proposed framework is able to retrieve all the patient data from iHealth and Google every 1 h by connecting to the respective vendor cloud using RESTful principles without requiring user intervention except for a one‐time credential authentication. In order for the system to connect to the iHealth API or the Google Fit API, authentication using the OAuth protocol is required [38]. OAuth 2.0 authorization protocol is the industry standard in authorization for web, desktop and mobile applications. This is the security protocol used for both the Google services, and iHealth API data interfacing. To mitigate the intrusiveness of prompting to authenticate themselves with the portal every time a medical professional or caregiver wants to monitor their data, a one‐time authentication technique is followed. The user logs into their iHealth account and Google account through the framework web portal at the time of initial registration to authorise access vendor cloud API permissions to the framework. This one‐time two‐step process is presented to the user on the web portal and they do not have to perform this action repeatedly. The web portal allows for the user input of optional variables such as skin fold thickness, number of pregnancies, and pre‐exising medical conditions if required. The unique user authentication token for each vendor API is obtained by the server and stored. The server connects to the respective vendor cloud API and automatically renews the token before its expiry dates to keep the token consistently updated. This is achieved by the setting up of a server‐side automated routine that triggers a token refresh function defined by the expiry parameters of each vendor API token.

### 4.2 Integration and visualisation node

The purpose of this node is to control the flow of data from the sensing node to the prediction node, address incomplete or duplicate data while providing medical professionals and the opportunity to observe the daily vitals and activity of users. To ensure security and interoperability, the cloud NoSQL database Google Firestore was used to establish communication of monitored data and authentication token between the mobile devices and the server. Using a trusted intermediary such as Google Firestore, auto‐managed cloud service allows the system to become relatively more secure than if security rules were custom implemented and deployed. The features of data syncing, backup and scaling is also obtained. The web portal was designed using HTML, CSS and Javascript using a front‐end framework VueJS. The portal is asynchronous in nature, therefore changes in user data are reflected on the portal immediately when viewed by medical professionals.

The list of available patient data in this framework and their respective sources are presented in Table 2. The server calculates the derived metrics by using the other variables that have already been collected, such as age, gender, height, and weight. The server aggregates readings on a daily basis, and orders the user data chronologically even within the day using the timestamp of data acquisition. Additionally, metrics such as total daily energy expenditure (TDEE), basal Metabolic rate (BMR) and BMI, are calculated to provide a holistic monitoring ability to the medical professionals.

**TABLE 2:** Data available on the portal and their sources

**Data**	**Source**
Height	Smartphone, smartwatch
Weight	Smartphone, smartwatch
Age	Smartphone, smartwatch
Step count	Smartphone, smartwatch
Total activity duration	Smartphone, smartwatch
Activity type	Smartphone, smartwatch
Calories	Smartphone, smartwatch
Glucose level	Glucometer
Medication status	Glucometer
Heart rate	Smartwatch, BP monitor, pulse oximeter
Systolic BP	BP Monitor
Diastolic BP	BP Monitor
Blood oxygen saturation	Pulse oximeter
Perfusion index	Pulse oximeter
BMI	Derived
BMR	Derived
TDEE	Derived
VO2Max	Derived
Diabetes risk	Predicted

For heart rate, maximum, average and minimum values are also estimated. The intention behind increasing the number parameters that can be derived from the acquired data is to facilitate the implementation of multiple predictive and monitoring techniques for accessible diabetic user lifestyle management.

**FIGURE 6:**
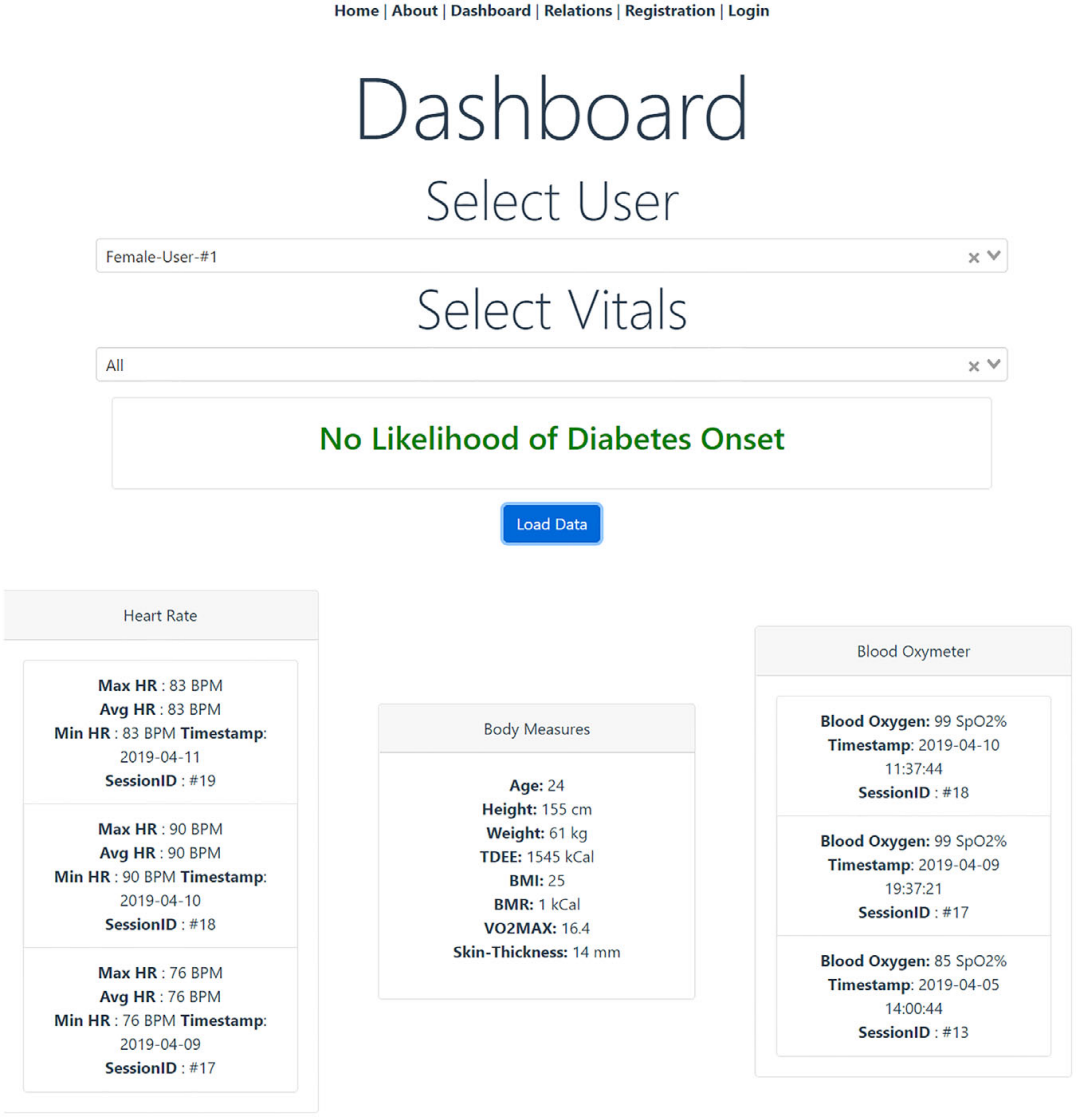
Doctor web portal showing patient data of heart rate, body measures and blood oxygen

**FIGURE 7:**
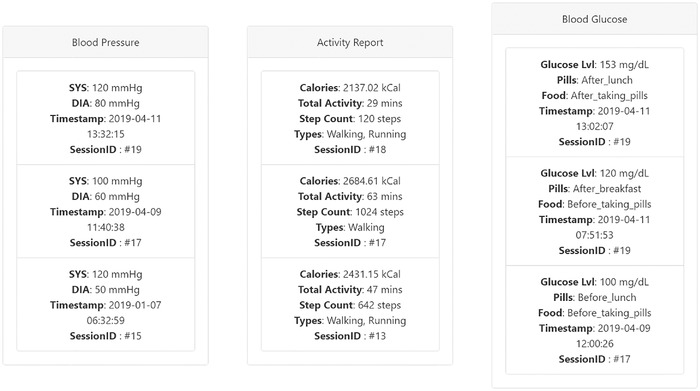
Doctor web portal showing patient data of blood pressure, activity report and glucose

Figure 6 and Figure 7 shows the medical history of the volunteer subject vitals for maximum heart rate, average heart rate, minimum heart rate, TDEE, BMI, BMR, blood oxygen saturation with their respective timestamps of acquisition. It also shows the history user vitals for systolic blood pressure, diastolic blood pressure, calories, total activity, number of steps taken, type of activity detected by smartphone/smartwatch, glucose level, pill and food status with the respective timestamps of acquisition. Prediction of diabetes is based on ML algorithms and a supplementary threshold based on user vitals. The input features extracted from the user measurements are the continuously monitored values of glucose. Age is updated based on their date of birth details provided through Google Fit. Serum insulin levels are a static value based on their last medical checkup with the provision to add any future devices that is capable of monitoring this value for improved reflection of patient states.

All the user vitals and measurements on the portal are updated automatically, provided that users adhere to regular use of their iHealth devices and smartphones. Experimental evidence shows that the development of diabetes can be delayed or even prevented if appropriate lifestyle changes are implemented [39]. Any auxiliary readings to be measured such as blood pressure, blood oxygen or activity metrics are automatically retrieved by the system and consolidated as part of the same session, during the same day. There can be multiple sessions with a unique ID per timestamp. The time window defining a session is 1 h as well, and isolated values if obtained are aggregated at the end of the day in accordance with the timestamp. This is done for harmonisation of user measurements, therefore medical professionals can see the effects of multiple factors that can lead to a positive diabetes class prediction by the system. The patient is required to take glucose measurements with the iHealth device as per the conditions based on the requirements of medical professionals. The conditions are before food; before pill, after food; before pill and after food; after pills.

Cloud privacy issues are a potential threat despite the resilient infrastructure of commercial cloud providers such as Google. To address this potential problem, a system generated hashed user ID is used to keep track of user data on cloud services. The hash function used the user metadata of patient profiles as key inputs and produced a 128 bit hash. The hashing function and key attributes were stored and made available only on the patient device and the doctor's system. Additional concerns of security are currently beyond the scope of this work. The holistic monitoring in the portal reflect factors relating to diet management, exercise, vital tracking and efficacy of pharmacological treatment. This can enhance the overall treatment process as medical professionals have these data conveniently available for risk stratification and making informed decisions.

### 4.3 Prediction node

In this node, the best performing ML algorithm among the four tested models is implemented for prediction of diabetes based on a subset collected user data. Figure 6 presents the machine learning diabetes prediction for subject in this work as “no likelihood of diabetes onset” based on the latest collected information from the framework. A new prediction is generated each time the respective predictor variables of glucose level, BMI, age, number of pregnancies, blood pressure and insulin are jointly updated. Alerts are provided to the medical professionals via the portal if there are multiple repeated diabetes positive results in a short time period for a single user from the models to enable actionable intervention from the professional side. This approach in addition to the other user vitals will allow the medical professionals to immediately identify the impact of risk factors such as low activity levels, weight gain, higher blood pressure, heart rates, glucose levels on the user's overall health.

## 5 RESULTS AND DISCUSSION

### 5.1 Experiment setting

The proposed framework was implemented on a workstation with Windows OS, an Intel Kabylake 2.80GHz processor (i7‐7700HQ) and 16 GB of RAM. The Python programming language was used for developing a Flask server to support the proposed framework and to develop the ML algorithms used in the prediction node. Additionally the Numpy and Pandas module was used for data pre‐processing and feature selection while the scikit‐learn module was used for configuring the ML algorithms [40].

### 5.2 Performance evaluation

The stratified *k*‐fold cross validation strategy was adopted to validate the performance variability of the model, and k=10 in this work. In each of the ten iterations, 3/5 parts of the data is used for training, 1/5 parts of the data is used for validation, and 1/5 of the data is used for testing. The final performance of the model is measured as the aggregate of results obtained on the testing set. To avoid data leakage and minimise model overfitting, MICE and SMOTE were only applied only the training and validation sets.

The performance measures considered for evaluating the ML models are accuracy, sensitivity and specificity and they are calculated by the formulas in Equation (7). These measures of diagnostic performance are selected as per standard ML assessment criteria in literature [41]. In this work, True positives (TP) are the number correct diabetes predictions, True negatives (TN) are the number of correct non‐diabetes predictions, false positives (FP) are the number of wrong diabetes predictions and false negatives (FN) are the number of wrong non‐diabetes predictions.

(7) $$\begin{array}{c} \text{Accuracy}=\frac{\text{TP}+\text{TN}}{\text{TP}+\text{TN}+\text{FP}+\text{FN}}\\ \text{Sensitivity}=\frac{\text{TP}}{\text{TP}+\text{FN}}\\ \text{Specificity}=\frac{\text{TN}}{\text{TN}+\text{FP}} \end{array}$$

Tables 3 and 4 summarise the best performing models along with their respective hyperparameters, which were identified using the GridSearch algorithm. The reported measures are the aggregates after performing stratified fivefold cross validation.

**TABLE 3:** Performance measures of ML models without preprocessing

**Model**	**Parameters**	**Acc%**	**Sen%**	**Sp%**
k‐NN	*k* = 5, weights=uniform	73.16	55.60	82.06
LR	*C*=1, tol=0.0001, reg=L2	76.40	49.63	90.80
NB	smoothing=0.000000001	75.5	61.0	82.80
SVM‐RBF	*C*=100, *g*=0.6	76.80	55.23	88.40

**TABLE 4:** Performance measures of ML models with feature selection, imputation and data augmentation

**Model**	**Parameters**	**Acc%**	**Sen%**	**Sp%**
k‐NN	*k* = 7, weights=uniform	79.80	87.20	72.40
LR	*C*=1, tol=0.0001, reg=L2	73.30	70.20	76.40
NB	smoothing=0.000000001	73.10	66.60	77.60
SVM‐RBF	*C*=5, *g*=0.4	83.20	87.30	79.00

There is a significant difference between in the sensitivity measures after performing feature selection and data augmentation. Training the model on the dataset without any preprocessing techniques as in Table 3 shows that there is high specificity in detecting cases that are not diabetes, however has a very low chance of detecting any diabetes cases. High sensitivity is pertinent as the system where this model is deployed is required to identify users who are at risk of developing diabetes for timely intervention. Therefore the trade‐off with higher diagnostic specificity for higher diagnostic sensitivity in this case can be justified.

Table 4 shows that the SVM‐RBF model achieves the highest accuracy, sensitivity and specificity. From these measures, the SVM‐RBF model can be considered as the best performing model in this work with the k‐NN being relatively close in performance. It is likely that more complex models such as ensemble models may be required for this particular dataset to effectively capture the underlying patterns.

**FIGURE 8:**
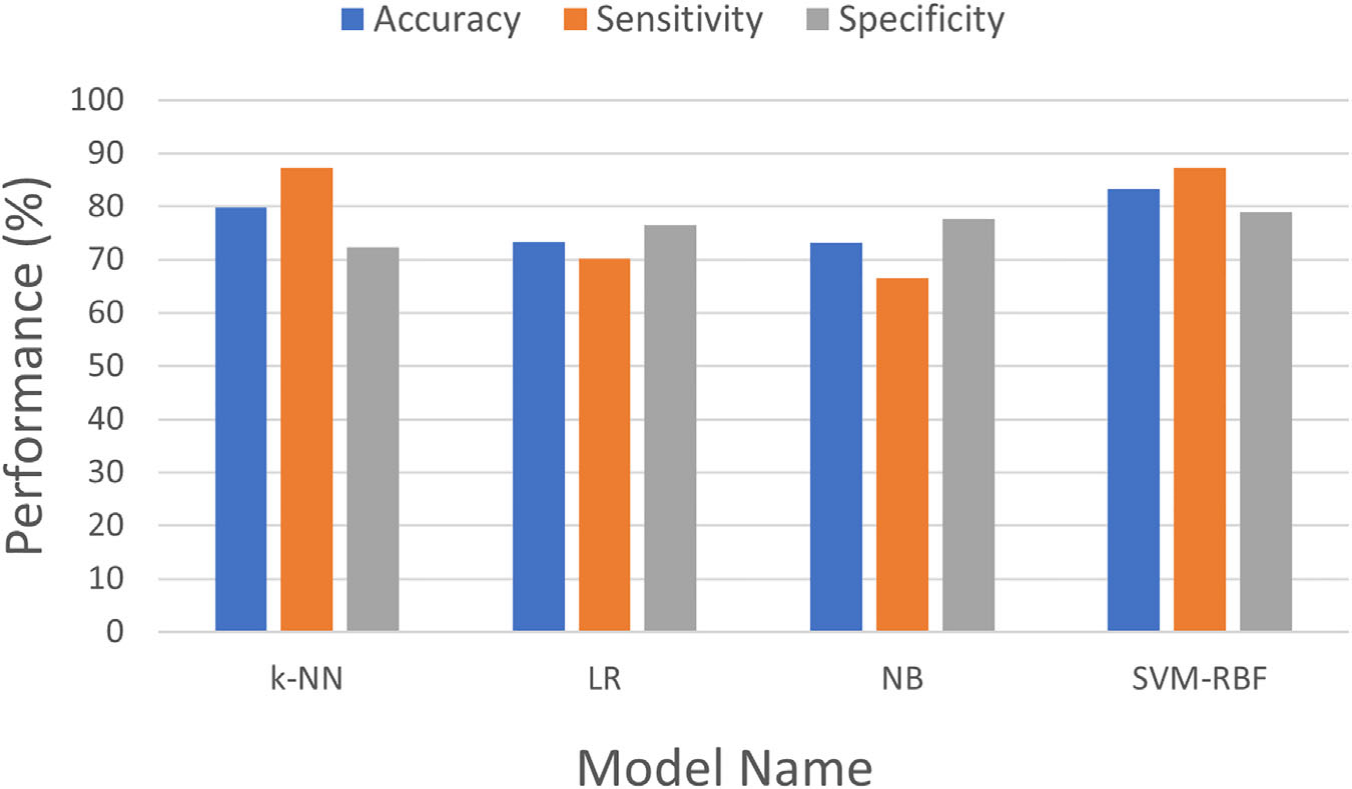
Performance measure comparisons

Figure 8 demonstrates the comparative results of the ML models with the respect to the performance measures.

### 5.3 Comparative study

Table 5 shows recent ML approaches in literature for prediction of diabetes using the PIMA Indian Diabetes Database. Only the performance measure of accuracy was consistent across the surveyed literature, as sensitivity, specificity or similar measures were not always reported.

**TABLE 5:** Comparisons with recent works in literature

**Author (year)**	**Best model**	**Acc%)**
**This work**	**SVM‐RBF**	**83.20**
Chatrati et al. (2020) [12]	SVM	75.00
Sonar et al. (2019) [13]	SVM	82.00
Al‐Zebari et al. (2019) [14]	LR	77.9
Saha et al. (2019) [15]	NN	80.4
Wei et al. (2018) [16]	Deep NN	77.86

The results obtained in this work are competitively similar to the works reported previously. The primary contribution of the paper is the novel approach for data acquisition from personal health devices and consumer smart devices, and its subsequent use for predicting diabetes risk. By closing the loop between disparate medical sensing device data, general purpose devices and isolated machine learning models, the aim is to present an alternate methodology for a smart healthcare monitoring and management system for chronic diabetes. A well‐known dataset is used, as it is considered a standard for diabetes prediction, and because all its predictor features can be obtained from easily accessible healthcare devices and ubiquitously present smartphones. This reduces friction when models trained on this dataset are to be used in practical scenarios. After an initial authorization step, the user is not required to manually input their vitals into the system, but can benefit from alerts regarding their diabetic state. Although clinical studies aimed at diagnosing diabetes has been growing recently, studies predicting the risk of developing diabetes is limited in an end‐to‐end system. Most of the existing works do not discuss the role of the developed model as part of framework where it can bridge the gap between patients and medical professionals, and as such is limited in their applicability and accessibility. This work is also one of the first automated diabetes management systems to provide an expanded view of all factors related to the physiological states of users, while having provisions for real‐time detection. The consolidation of information from multiple devices solves vendor interoperability issues, and relevant information about users is made available to medical professionals.

The main limitations of this work arise due to the narrow scope of the dataset and small‐scale testing of the entire system. The dataset included only samples of women of Pima Indian descent, thereby introducing gender and ethnicity bias. While these attributes were not selected as features in the feature selection step for training our model, underlying association may lower generalisability. Consequently, this system was tested only with two female volunteers and one medical supervisor, and had achieved expected results (i.e. one case of diabetes and one case of non‐diabetes). A longitudinal study with a larger cohort will be the focus of a follow up research, and its details are beyond the scope of this work, which presents initial implementation results of a complete system.

## 6 CONCLUSION

This paper proposed a framework that automates diabetes detection and alerts medical professionals for timely intervention. The developed system integrated multiple healthcare devices and consumer devices using cloud principles and provided the acquired readings to medical professionals for enabling improved diagnostic decision making. Four supervised ML algorithms were implemented, and the best performing one was deployed on the proposed framework. This was experimentally revealed to be the SVM‐RBF, which achieved an accuracy of 83.20 %, sensitivity of 87.20%, and specificity of 79%. The major contribution of this work is the implementation of a diabetes prediction model into an RPM framework that is modular, multi‐faceted, and requiring minimal patient interaction with the platform. The system is vendor‐independent and interoperable to ensure eventual addition of new prediction models and personal health care devices for improving patient outcomes. Future research directions involve conducting a longitudinal study with a larger cohort of patients and subsequent testing of the system with integration of additional devices and patient information.
